# Yorkshire Enhanced Stop Smoking (YESS) study: a protocol for a randomised controlled trial to evaluate the effect of adding a personalised smoking cessation intervention to a lung cancer screening programme

**DOI:** 10.1136/bmjopen-2020-037086

**Published:** 2020-09-10

**Authors:** Rachael L Murray, Kate Brain, John Britton, Harriet D Quinn-Scoggins, Sarah Lewis, Grace M McCutchan, Samantha L Quaife, Qi Wu, Alex Ashurst, David Baldwin, Philip A J Crosbie, Richard D Neal, Steve Parrott, Suzanne Rogerson, Rebecca Thorley, Matthew EJ Callister

**Affiliations:** 1 Division of Epidemiology & Public Health, Faculty of Medicine, University of Nottingham, Nottingham, United Kingdom; 2 UK Centre for Tobacco and Alcohol Studies, University of Nottingham, Nottingham, United Kingdom; 3 Division of Population Medicine, Cardiff University, Cardiff, United Kingdom; 4 Research Department of Behavioural Science and Health, University College London, London, United Kingdom; 5 Department of Health Sciences, University of York, York, UK; 6 Department of Radiology, Leeds Teaching Hospitals, Leeds, United Kingdom; 7 Deaprtment of Respiratory Medicine, Nottingham University Hospitals NHS Trust, Nottingham, United Kingdom; 8 Division of Infection, Immunity and Respiratory Medicine, The University of Manchester, Wythenshawe, UK; 9 Institute of Health Science, University of Leeds, Leeds, United Kingdom; 10 Research and Innivation CSU, Leeds Teaching Hospitals, Leeds, United Kingdom; 11 Department of Respiratory Medicine, Leeds Teaching Hospitals, Leeds, United Kingdom

**Keywords:** protocols & guidelines, public health, CT

## Abstract

**Introduction:**

Integration of smoking cessation (SC) into lung cancer screening is essential to optimise clinical and cost effectiveness. The most effective way to use this ‘teachable moment’ is unclear. The Yorkshire Enhanced Stop Smoking study will measure the effectiveness of an SC service integrated within the Yorkshire Lung Screening Trial (YLST) and will test the efficacy of a personalised SC intervention, incorporating incidental findings detected on the low-dose CT scan performed as part of YLST.

**Methods and analysis:**

Unless explicitly declined, all smokers enrolled in YLST will see an SC practitioner at baseline and receive SC support over 4 weeks comprising behavioural support, pharmacotherapy and/or a commercially available e-cigarette. Eligible smokers will be randomised (1:1 in permuted blocks of random size up to size 6) to receive either an enhanced, personalised SC support package, including CT scan images, or continued standard best practice. Anticipated recruitment is 1040 smokers (January 2019–December 2020). The primary objective is to measure 7-day point prevalent carbon monoxide (CO) validated SC after 3 months. Secondary outcomes include CO validated cessation at 4 weeks and 12 months, self-reported continuous cessation at 4 weeks, 3 months and 12 months, attempts to quit smoking and changes in psychological variables, including perceived risk of lung cancer, motivation to quit smoking tobacco, confidence and efficacy beliefs (self and response) at all follow-up points. A process evaluation will explore under which circumstances and on which groups the intervention works best, test intervention fidelity and theory test the mechanisms of intervention impact.

**Ethics and dissemination:**

This study has been approved by the East Midlands-Derby Research Ethics Committee (18/EM/0199) and the Health Research Authority/Health and Care Research Wales. Results will be disseminated through publication in peer-reviewed scientific journals, presentation at conferences and via the YLST website.

**Trial registration numbers:**

ISRCTN63825779, NCT03750110.

**Strengths and limitations of this study**

- This is the first study to investigate the use of incidental findings as part of a smoking cessation (SC) intervention delivered alongside lung cancer screening, and to evaluate the uptake and effectiveness of a colocated stop smoking service.
- Recruitment is limited to the number of eligible smokers attending for a lung health check as part of the Yorkshire Lung Screening Trial.
- SC support will be consistently delivered in line with National Health Service best practice and National Institute for Health and Care Excellence guidance.
- The scripted approach to explaining lung scan results will support standardisation of intervention delivery.
- The inclusion of a process evaluation will enhance contextual understanding of study outcomes, including why eligible individuals do not engage with the SC intervention.

## Introduction

Lung cancer is the third most common cancer and has the highest mortality of all cancers in the UK.^1^ It becomes more common as socioeconomic deprivation levels increase,^2^ reflecting higher smoking rates.^3^ A reduction in both all-cause and lung cancer specific mortality was reported by the US National Lung Screening Trial in 2011^4^; subsequently lung cancer screening (LCS) was adopted across North America having been recommended by the US Preventive Services Task Force in 2013.^5^ Mortality benefits of low-dose CT (LDCT) screening for LCS have subsequently been demonstrated by the Multicentric Italian Lung Detection (MILD) trial^6^ and confirmed by preliminary results from the NELSON study.^7^

More than 85% of cases of lung cancer are caused by tobacco smoking,^8^ and stopping smoking at any age significantly reduces lung cancer risk.^9 10^ LCS may provide a teachable moment for smoking cessation (SC);^9^ patients undertaking routine or acute visits to healthcare providers are more likely to be receptive to offers of help to quit.^11^

Several large studies have measured SC outcomes in the context of LCS, with results varying between higher quit rates,^12^ no difference^13^ or lower quit rates in screened versus unscreened participants.^14^ Despite the potential for important health gains, there is limited evidence on how best to integrate effective SC services in an LCS context, though lessons may be learnt from SC interventions delivered in other settings.

Evidence suggests that provision of SC support as an opt-out default generates more quit attempts,^15–17^ and higher uptake within an LCS setting.^18^ A 2014 systematic review showed benefit of materials tailored to the characteristics of individual smokers,^19^ although the included studies were conducted predominantly in the general population, rather than screening participants. Further, a recent UK study has demonstrated the efficacy of including risk information personalised to pre-existing health conditions and consequences of continuing to smoke when inviting participants to SC services.^20^ LDCT screening commonly detects smoking-related comorbidities, a consequence of participants’ long smoking histories.^21 22^ The screening process is a unique opportunity for a personalised SC intervention using these findings to improve quit rates.^23^

The SCALE collaboration in the USA consists of eight ongoing projects, and has recently been established to support projects testing a variety of SC interventions delivered in LCS settings involving LDCT. All share a common core of data collection measures to facilitate data sharing, which will build an evidence base for effective approaches.^24^ One approach not being tested within the SCALE collaboration is the use of incidental scan findings as part of the SC intervention. The Yorkshire Enhanced Stop Smoking (YESS) study intervention consists of a personalised paper-based booklet intervention incorporating images of the heart and lungs from the participant’s own LDCT scan. The booklet will be delivered by an SC practitioner (SCP) specially trained to deliver the intervention components, highlighting the short-term and long- term benefits of cessation while boosting self and response efficacy. To our knowledge, the YESS study is the first to test such an approach.

### Study aims

The primary aims of the study are to:

1. Assess the uptake and effectiveness of a colocated opt-out SC intervention, delivered in line with National Health Service (NHS) standard best practice (SBP) guidance, as part of an LCS programme.
2. Assess the efficacy of a personalised, theory-grounded SC intervention, incorporating participant heart and lung scan images alongside an explanation of the clinical importance of the findings and scripted communication to enhance personal salience, self-efficacy and response efficacy.

The secondary aims of the study are:

1. Conduct a process evaluation of both the colocated and personalised SC intervention to support interpretation of study findings.
2. Conduct a health economic evaluation to assess the cost-effectiveness of the colocated service plus personalised feedback intervention over and above the colocated service plus SBP.

## Methods

**Figure 1:**
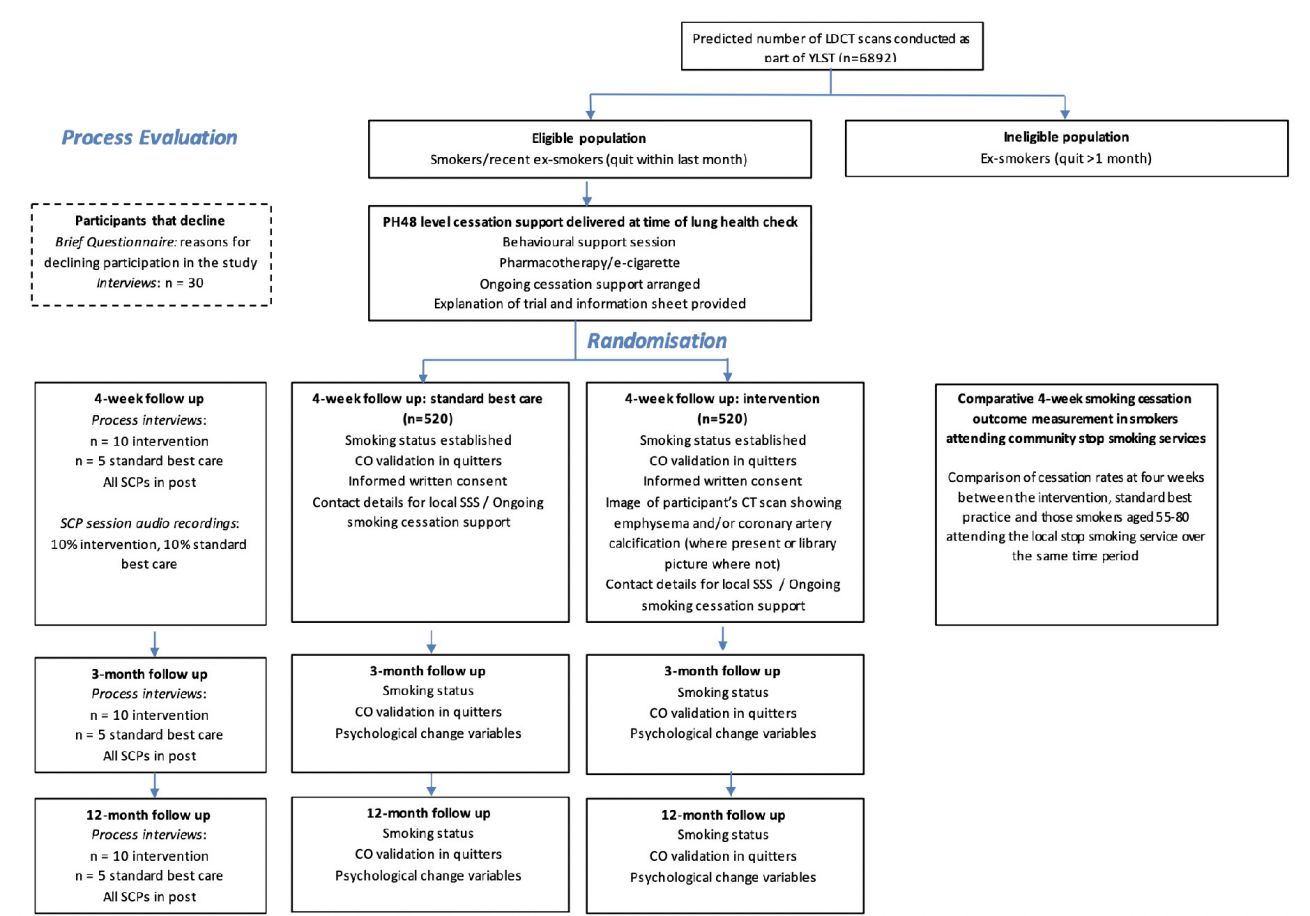
Flow diagram illustrating the path of participants through the study, interventions and evaluations. LDCT, low-dose CT; SCP, smoking cessation practitioner; YLST, Yorkshire Lung Screening Trial; SSS, stop smoking service

This protocol is reported in accordance with Standard Protocol Items: Recommendations for Interventional Trials guidance.^25^ Participant flow and study measures are summarised in figure 1.

### Trial design

This is a pragmatic, parallel-group, open, randomised controlled trial comparing an enhanced, personalised SC support package, tailored to include results from LCS, delivered in addition to an SBP cessation package, versus SBP package alone. Further, we will also compare the effectiveness of the colocated service with local stop smoking services (SSS) in an observational design.

### Participants and recruitment

Participants included in the YESS study will have been invited to attend a lung health check (LHC) (including LDCT screening for lung cancer) as part of the Yorkshire Lung Screening Trial (YLST) (funded by Yorkshire Cancer Research, award reference L403, 2016 round REC reference 18/NW/0012).^26^ In brief, participants are aged 55–80, registered with a general practitioner (GP) in the Leeds Clinical Commissioning Group area and registered as a current or ex-smoker in primary care databases. Participants will be considered eligible for LDCT screening if they meet the criteria of either USPSTF,^27^ PLCO_M2012_ 28 or LLP (v2)^29^ models.

### Inclusion criteria

All individuals who attended an LHC and consent to participate in the YLST, have smoked within the last month or have an exhaled carbon monoxide (CO) reading ≥6 ppm and have agreed to see an SCP on the mobile unit.

### Exclusion criteria

Any individual who does not have an LDCT scan, or is unable to provide informed consent.

### Participant withdrawal

Participants may be withdrawn from the trial either at their own request or at the discretion of the Investigator. Participants will be made aware that this will not affect their future care.

### Randomisation

After the screening visit, but before the 4-week follow-up, all smokers who agreed to see the SCP will be randomised to either continued SBP or intervention using concealed allocation. The randomisation sequence (1:1 in permuted blocks of random size up to size 6) will be generated using a computer random-number generator and participants allocated sequentially, overseen by the University of Nottingham clinical trials unit.

### Blinding

The sequence of treatment allocations will be concealed until interventions have all been assigned and recruitment and data collection are complete. Once primary and secondary data from both treatment groups have been analysed by the trial statistician, the groups will be un-blinded to the Trial Management Group (TMG). Consent is taken at the 4-week visit by the SCP. In order to ensure concealment at the time of recruitment/consent, the personalised risk information booklet (further detail provided later) or a blank booklet are enclosed in a sealed envelope which is opened by the SCP following consent/data collection. This arrangement commenced five rounds into recruitment following review of processes at a Trial Steering Committee. For five rounds at the start of the trial, SCPs were aware of trial allocation at the time of consent/data collection. The impact of this will be assessed in a sensitivity analysis.

### Sample size

Recruitment will be limited to the number of eligible smokers attending for an LHC over the 2-year YLST recruitment period. Based on early recruitment figures, we anticipate 1040 smokers enrolling in YESS.

Those in the SBP group will be provided with a service similar to that provided by national NHS SSS. A 2010 systematic review of relapse rates among smokers quitting with national NHS SSS found that 53% had quit at 1 month, and 15% at 1 year.^30^ Fitting an exponential decay function to these figures we estimate a monthly relapse rate, after 1 month, of 11.5%. Applying these figures to our study design we would expect a quit rate of 41% at 3 months in those receiving SBP. If the cessation rate is 41% in the usual care group, a study of 1040 individuals will provide 90% power to detect an increase to 51% (ie, an increase of 10%), and 80% power to detect an increase to 49.6% (ie, an increase of 8.6%).

### Study duration

Participants will be recruited to YESS between January 2019 and December 2020 with follow-up data collection ending December 2021. Stop smoking support will be provided for as long as the participant requires, up to 12 weeks. Follow-up contact will be requested at 4 weeks, 3 months and 12 months, with a 2-week window to accommodate participant availability. The latter will also apply to interviews conducted for the process evaluation.

### SC provision

#### All eligible smokers

Unless explicitly declined, all eligible YLST participants will attend a consultation with a specialist SCP, trained to National Centre for Smoking Cessation and Training standards,^31^ colocated within the screening van. Support will be provided in line with National Institute for Health and Care Excellence (NICE) PH48 guidance^32^ comprising one session of behavioural support at the time of the LHC and provision of pharmacotherapy (either as nicotine replacement therapy through delegated prescribing at the visit and/or a commercially available e-cigarette, or arrange a GP prescription for varenicline or bupropion). Follow-up contact either face-to-face or by telephone, typically weekly but more or less frequently according to participant preference for up to 4 weeks from the date of the LHC.

Where study SCP follow-up is not possible, or if the individual smoker prefers, contact details will be passed to the local NHSSSS for referral into community services immediately following the screening visit.

All eligible participants will be asked for consent to be contacted by telephone at 4 weeks, 3 months and 12 months after the screening visit solely to ascertain smoking status, with optional CO validation if quit. Approximately 4 weeks after the screening visit (and hence after scan results have been issued by YLST), the SCP will arrange a face-to-face visit with the smoker either at home or community location (according to the participant’s preference) to ascertain current smoking status (with CO validation in those reporting abstinence). Informed written consent for participation in the trial will be confirmed at this visit, and individuals treated according to their allocated group as follows:

#### SBP group

Pharmacotherapy/e-cigarettes and behavioural support will continue to be offered and arranged, as outlined above.

#### Intervention

##### Personalised booklet with LDCT scan images

In addition to SBP and YLST standard feedback, participants are provided with information about their scan findings in a personalised booklet. Where the reporting radiologist has identified emphysema or coronary artery calcification (CAC), a trained research radiographer will select appropriate images for use in the booklet. All images are accompanied by brief text to provide context and explanation.

**Figure 2:**
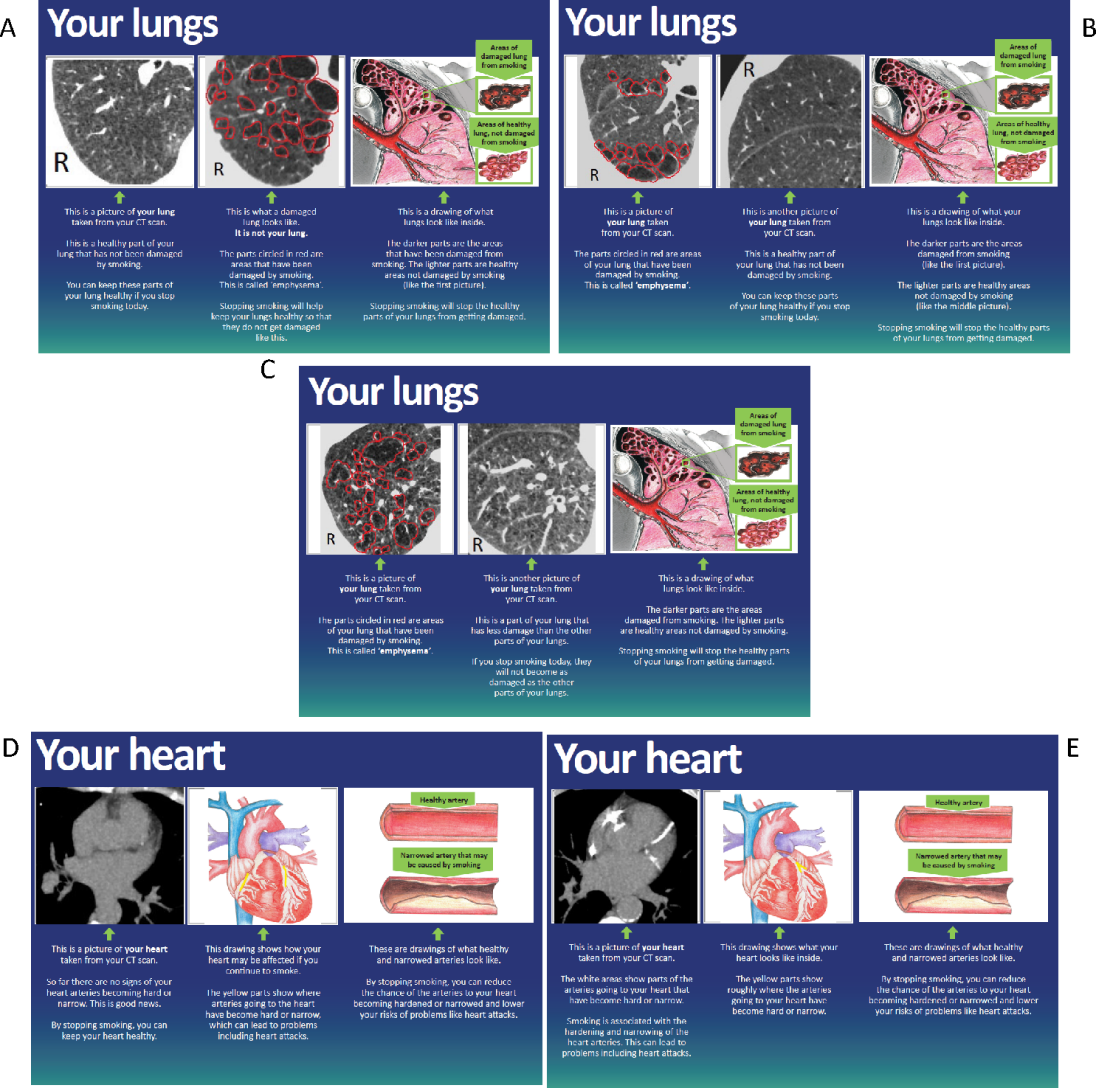
Example of a booklet page for (A) participant with no visible emphysema; (B) participant with ‘moderate’ emphysema; (C) participant with ‘severe’ emphysema; (D) participant with no visible coronary artery calcification (CAC); (E) participant with CAC.

Where the participant’s scan shows emphysema, an axial image displayed using lung windows that best demonstrates this will be extracted, and a red outline drawn around the emphysematous area to highlight this finding. Where possible, an image from another part of the participant’s scan showing normal lung parenchyma will be shown alongside the emphysematous image to highlight that there are areas of healthy lung than can be protected by SC. Where emphysema is present throughout both lungs, an image will be selected showing an area of relative sparing. Where emphysema is not present at all, an image of the participant’s normal lung will be shown alongside a library scan image of severe emphysema. An artists’ impression of a lung, showing healthy and damaged emphysematous areas will be included to aid participant comprehension (see figure 2A–C, for examples).

Where CAC is present, the axial image displayed using mediastinal windows that best demonstrates this will be extracted and shown alongside an artist’s impression of the heart, with the corresponding area of calcification annotated with yellow shading. Where CAC is not present, the participants’ own normal scan image will be shown alongside an artist’s impression of the heart demonstrating what might happen were the participant to continue smoking. In all cases, an additional artist’s impression of health and narrowed coronary arteries are shown (see figure 2D, E, for examples). Five per cent of all booklets produced will be audited by a respiratory physician or radiologist to ensure that appropriate images, annotations and text are used in the resource.

The booklet will also contain generic short-term and longer-term benefits of quitting derived from the Smokefree NHS website^33^ and contact details for the YESS study project manager, the local NHSSSS, the YLST clinical team and the assigned SCP.

##### Scripted communication to explain clinical importance and target behavioural components

The SCP will explain the biological nature of emphysema and CAC and will provide feedback and further SC advice tailored to the result. The SCP will highlight specific benefits to risks of lung cancer, Chronic Obstructive Pulmonary Disease (COPD) or cardiovascular disease arising from quitting including the reduced progression of existing emphysema or smoking-damage. SCP’s communication about the personalised risk booklet is supported by scripts which exemplify how each section should be communicated in each of the different result scenarios. The scripts model communication techniques and language designed to target the behavioural intervention components; specifically, to increase personal salience, self-efficacy and response-efficacy, while mitigating the potential for anxiety and over-reassurance. They use lay language to illustrate the steps of disease progression and emphasise the short-term and immediate benefits of quitting for participants’ health. This scripted approach also aims to help standardise intervention delivery. Where required, participants are offered the opportunity for a telephone consultation with a respiratory physician to discuss scan findings.

### Data collection

Data collection measures are summarised in table 1.

**Table 1:** Data collection measures in study participants

	Baseline	4 weeks	3 months	12 months
Smoking behaviour (including CO validation if abstinence is reported)	X	X	X	X
Motivation to quit smoking	X	X	X	X
Quit confidence	X	X	X	X
Self-efficacy of quitting smoking	X	X	X	X
Use of smoking cessation support	X			X
Wider healthcare resource use	X		X	X
Response efficacy of quitting smoking		X	X	X
Perceived risk of cancer developing cancer		X	X	X
Cancer worry score		X	X	X
Quality of life (EQ-5D-5L)	X		X	X

CO, carbon monoxide.

### Outcome measures

#### Primary outcome

Seven day point prevalent CO validated SC 3 months after the LHC in all participants enrolled in the YESS study.

#### Secondary outcomes

- Self-reported and CO validated continuous cessation at 4 weeks in all eligible smokers agreeing to receive SC from the SCP as part of the LHC.
- Self-reported continuous and seven day point prevalent SC at 3 months.
- Self-reported and CO validated continuous cessation at 12 months.
- Attempts to quit smoking.
- Changes in psychological variables, including perceived risk of lung cancer, motivation to quit smoking tobacco, confidence and efficacy beliefs (self and response) at all follow-up points.

### Process evaluation

Approximately 30 semistructured interviews (10 at each follow-up time point) with be conducted with intervention participants, or until data saturation, to evaluate intervention usage, comprehension and acceptability. Fifteen SBP group participants (five at each time point) will be interviewed to assess potential study contamination. Interviews will also explore whether participants received any additional risk information or SC advice from healthcare professionals as part of their LHC or during any nodule follow-up. Participants will be purposively sampled by age (≤65 years, >65 years), gender, quit motivation, quit status (at 3 and 12 months) and scan findings (ie, CAC present or absent; emphysema absent, present with areas of normal lung elsewhere or diffusely present throughout both lungs) across all interview time-points where possible. Interviews will be conducted across the 2-year trial recruitment timeframe, face-to-face or by telephone according to preference. Participants will be offered a shopping voucher as an inconvenience allowance.

All SCPs in post will be invited to take part in an interview across the three follow-up time points to explore which aspects of the intervention were perceived to influence change, gain insight into contextual barriers and facilitators of embedding the personalised intervention into practice and intervention fidelity.

Audio-recordings of SCP sessions will take place (10% across both arms) across the 2-year recruitment period to assess intervention fidelity. Sessions to be recorded will be purposefully sampled by SCP, quit motivation (where possible), age (≤65 years, >65 years) and gender (men/women). Fidelity will be ascertained by a trained researcher listening to the audio-recordings and coding the appropriate delivery of materials against SBP and standard care plus personalised risk information. To complement session audio-recordings and SCP interviews, SCPs will be asked to self-report fidelity (intervention elements were delivered as intended) and dose (how much of the intervention was delivered).

All interviews will be audio-recorded using an encrypted recorder with permission, transcribed verbatim and anonymised. The audio recording will be labelled using the participant’s unique identification number assigned during YLST and transferred to an external transcribing service. Audio-files will be destroyed after analysis is complete.

### Economic evaluation

An economic evaluation will assess the cost-effectiveness of the personalised feedback intervention over and above standard care. An NHS perspective will be adopted in line with NICE guidance. We will record and calculate the costs of providing the colocated service, the SBP and the intervention prospectively throughout the trial, in terms of staff time, overheads, pharmacotherapy and other consumables. We will also record patients’ utilisation of primary and secondary healthcare services outside of the trial using a short self-report questionnaire. The primary health outcome in the economic evaluation is assessed in terms of quality-adjusted life years (QALYs). The EuroQoL EQ-5D-3L questionnaire will be used to measure health-related quality of life and calculated QALYs using the area under the curve approach.^34^ Questionnaires will be administered at baseline, 3-month and 12-month follow-up.

#### Non-participants

Eligible participants who attend for an LHC but opt-out of receiving SC support, or receive SC support but do not consent to participate in the YESS study will be asked to complete a brief questionnaire detailing their reasons for declining. Further, individuals who receive SC support but do not consent to participate in the YESS study will be asked to provide consent to be contacted at 3 and 12 months to ascertain smoking status only.

Additionally, we will purposively sample approximately 30 non-participants by quit motivation (where possible), age (≤65 years, >65 years) and gender for interview to understand their reasons for non-participation. Interviews will be carried out face-to-face or by telephone, according to preference, and participants will be offered a shopping voucher. Where possible interviews will be conducted within a 2-week window after declining. We will conduct interviews across the 2-year trial recruitment timeframe. Consent for interviews with those who decline will be obtained via telephone according to a standardised consent script. Information sheets and a copy of the consent script will be sent to participants prior to interview.

#### Comparative measurement in those smokers attending community stop smoking services

Anonymised data will be provided for those aged 55–80 years for dates corresponding to the study period from the local NHSSSS to compare quit outcomes in those receiving SC support through YESS with those accessing community services.

### Assessment of adverse events

Adverse events arising during the trial will be managed in accordance with standard clinical practice, recorded by the trial manager. Electronic cigarette use by participants will be recorded and participants will be able to report their experience at the 4-week, 3-month and 12-month follow-up assessments.

### Data management and monitoring

Monitoring of trial data shall include: confirmation of informed consent; source data verification; data storage and data transfer procedures; local quality control checks and procedures, back-up and disaster recovery of any local databases and validation of data manipulation. Entries on case report forms (CRF) will be verified by inspection against the source data. A sample of CRFs (10% or as per the study risk assessment) will be checked on a regular basis for verification of all entries made. In addition, the subsequent capture of the data on the trial database will be checked. Interview transcripts will be checked against audio-recordings for accuracy. Where corrections are required these will carry a full audit trail and justification. Trial data will be stored and maintained on Nottingham or Cardiff University respective servers. When and if data are transferred it will be conducted in a controlled/verified manner in accordance with a data management plan and the University of Nottingham’s ‘Handling Personal Data’ policy.

### Statistical analyses

We will compare baseline characteristics between randomised treatment groups descriptively. We will estimate the effect of the intervention by comparing our primary and secondary outcomes between intervention and SBP groups using logistic regression, presenting results in terms of the proportion achieving abstinence in the two groups, the risk difference and the OR, with 95% CIs.

Primary analysis will be on an intention to treat basis, assuming that missing data implies smoking, but we will conduct sensitivity analysis to examine the impact on our findings of alternative assumptions, including the Hedeker approach which assumes alternative relationships between having missing data and smoking, and using multiple imputation to test the alternative assumption that the data are missing at random (ie, missing data can be modelled using measured variables).

We do not plan a priori to adjust for any baseline covariate, presuming that randomisation will achieve balance between groups. We will look for effect modification of effect of intervention according to presence of absence of abnormal scan findings of emphysema or CAC, using the likelihood ratio test for interaction. In sensitivity analysis, we will analyse excluding those who were consented to the intervention group prior to change in timing of consent (n=94).

#### Comparative measurement in those smokers attending community stop smoking services

We will compare cessation rates at 4 weeks between the intervention and SBP groups with those smokers aged 55–80 attending the local stop smoking service over the same time period to delineate the effect of both SC interventions within YESS, using logistic regression and adjusting for observed baseline differences in predictors of cessation including age, gender, heaviness of smoking and level of education.

A detailed statistical analysis plan will be prepared prior to completion of trial recruitment, and agreed with the trial steering committee.

### Economic analysis

An incremental cost-effectiveness analysis will be conducted alongside the trial to assess the cost-effectiveness of the personalised feedback intervention over and above standard on an intention-to-treat basis. An NHS perspective will be adopted in line with NICE guidance.^35^ Costs will be calculated using national average unit costs multiplying the reported level of healthcare resource use. A total healthcare cost profile for all patients in each trial arm is then constructed by adding the costs of the intervention and standard care to the wider NHS costs. The economic evaluation will use validated quit rates at 12 months to estimate the incremental cost per QALY afforded by the intervention over and above SBP. The results presented will indicate the NHS cost of funding the intervention in order to gain one additional QALY at 12 months.

To handle the problem of missing trial data, we will conduct a sensitivity analysis employing Rubin’s multiple imputation method.^36^ Thirty multiple imputation (MI) data sets will be generated using the Multiple imputation (MI) command in STATA v.16. The outcomes of the sensitivity analysis will be used to test the impact of missing data on the results. To account for uncertainty due to sampling variation in cost-effectiveness, we will undertake a non-parametric bootstrapping on the incremental cost and effectiveness with 50 000 replications.^37^ Cost-effectiveness acceptability curves will be plotted based on the bootstrap iterations to show the probability of the intervention being more cost-effective than the standard care over a range of a decision-maker’s willingness-to-pay thresholds.^38^

### Process evaluation analysis

The process evaluation will be reported in line with Enhancing the QUAlity and Transparency Of Health Research guidelines.^39^ Anonymised interview transcripts will be analysed using a Framework approach^40^ with the aid of NVivo software^41^ or Microsoft Excel V.16 where appropriate for indexing. Themes will be derived initially from the process evaluation aims and topic guides, with additional themes emerging from the data. The analytic framework will be refined by double-coding 20% of the interviews. Analysis of intervention and SBP, trial non-participant and SCP interviews will be conducted separately, with comparisons made between the themes identified within the respective analyses. If the Motivation to Stop Smoking scale^42^ is used during interviews with non-participants, the response will be recorded where appropriate and a descriptive analysis will be conducted for interpretation alongside the qualitative interview data.

Audio-recordings will be analysed for context, fidelity and exposure. Fidelity will be assessed through the coding of intervention components delivered against those pre-set for standard care or standard care plus personalised risk information. Coding will take place on a standardised proforma (developed prior to study commencement), and a fidelity definition and acceptable range will be agreed on in advance. Twenty per cent of audio-recordings will be double-coded independently (10% of recordings across both arms). Descriptive statistics will be used to report intervention fidelity.

Closed-ended questions from SCP self-report data will be analysed using descriptive statistics to assess intervention fidelity and dose, using Microsoft Excel V.16 or SPSS V.26.^43^ Open-ended questions will be thematically analysed using NVivo.^41^ Free text data from non-participant questionnaires will be thematically analysed^44^ using NVivo, with 20% double-coded.

Trial data used to support the process evaluation will be analysed in line with the YESS trial statistical analysis plan. Descriptive analysis will be used to ascertain trial recruitment and retention and add to qualitative data on context, reach and exposure.

### Ethical approval, research governance, trial sponsorship and registration

This study was approved by the East Midlands-Derby Research Ethics Committee (REC reference: 18/EM/0199) and HRA/HRCW on 31 August 2018. Subsequent amendments are detailed in table 2.

**Table 2:** Summary of ethical amendments

Protocol	Date	Summary of changes
V2	16 October 2018	Re-formatting of consent forms to fit within one A4 page (minor).
V2.1	29 March 2019	Amendment to letters informing/seeking guidance from participants’ general practitioner regarding potential contraindications for nicotine replacement therapy and requesting varenicline prescriptions.
		Minor amendments to interview topic guides.
		Change to incentive amounts.
		Audio recording of a sample of initial consultation interactions (substantial).
V2.2	03 June 2019	Change timing of consent to YESS trial—not implemented.
V2.1	26 July 2019	Submission of YESS subsection 1 and 2 participant information sheet (V.1 17 05 2019) as they were omitted from the original application (substantial).
V2.2	11 November 2019	Reduction in recruitment target from 2019 to 1040 to reflect the number of smokers enrolling in YLST (substantial).

YESS, Yorkshire Enhanced Stop Smoking; YLST, Yorkshire Lung Screening Trial.

This study was adopted onto the National Institute for Health Research trial portfolio on 18 September 2018 and is sponsored by the University of Nottingham. Any planned modifications to the protocol will be approved by the REC before they are adopted by the study. An audit trail of ethical amendments, documentation and data collection will be kept to allow monitoring by the research team and external regulatory bodies.

The trial was registered with the International Standard Randomised Controlled Trial Number (ISRCTN) on 25 September 2018 and the National Institutes of Health ClinicalTrials.gov database on 21 November 2018.

### Study management

The TMG is comprised the principal investigator, clinical and academic coapplicants and collaborators who will jointly monitor trial conduct and progress. All aspects of the study, and study personnel, will adhere to the full clinical trial protocol (version 2.2 or subsequent approved version), Good Clinical Practice guidelines and General Data Protection Regulations.

A Trial Steering Group comprising independent experts in the fields of cancer screening, respiratory medicine, SC, radiology and statistics, and a patient/public representative will meet with key members of the TMG at approximately 6-month intervals throughout the trial recruitment phase to oversee this study and agree any amendments to the protocol.

## Discussion

The YESS study will address a number of questions directly relevant to the implementation of a smoking cessation intervention within LCS services. It will provide evidence as to the uptake, effectiveness and acceptability of an SC service colocated within a screening programme, and the efficacy and acceptability of providing a personalised stop smoking intervention which incorporates incidental findings detected as part of the LDCT scan supported by communication that supports self-efficacy and response-efficacy. Given the current interest in LCS programmes and the potential for adoption and implementation in many countries, the YESS study is timely and will potentially inform related policy decisions and recommendations.

Study findings will be written in accordance with Consolidated Standards of Reporting Trials guidelines,^45^ submitted for publication to relevant peer-reviewed journals, presented at conferences and published on the relevant section of the YLST website. A summary of results will be provided to any participant on request.

## Supplementary Material

Reviewer comments
