# Supplementary material for: Yorkshire Enhanced Stop Smoking (YESS) study: a protocol for a randomised controlled trial to evaluate the effect of adding a personalised smoking cessation intervention to a lung cancer screening programme
Source: BMJ Open. 2020 Sep 10;10(9):e037086. doi: 10.1136/bmjopen-2020-037086 (PMC7485260; doi:10.1136/bmjopen-2020-037086)
Supplement: Reviewer comments [file bmjopen-2020-037086.reviewer_comments.pdf]

PEER REVIEW HISTORY

BMJ Open publishes all reviews undertaken for accepted manuscripts. Reviewers are asked to complete a checklist review form (**http://bmjopen.bmj.com/site/about/resources/checklist.pdf**) and are provided with free text boxes to elaborate on their assessment. These free text comments are reproduced below.

ARTICLE DETAILS

**TITLE (PROVISIONAL)**	The Yorkshire Enhanced Stop Smoking study (YESS): a protocol for a randomised controlled trial to evaluate the effect of adding a personalised smoking cessation intervention to a lung cancer screening programme
**AUTHORS**	Murray, Rachael; Brain, Kate; Britton, John; Quinn-Scoggins, H; Lewis, Sarah; McCutchan, GM; Quaife, Samantha; Wu, Qi; Ashurst, Alex; Baldwin, David; Crosbie, Philip; Neal, Richard; Parrott, Steve; Rogerson, Suzanne; Thorley, Rebecca; CALLISTER, Matthew

This article was not externally reviewed at BMJ Open. Protocols that have been independently assessed prior to submission to BMJ Open are usually fast-tracked to publication on the grounds that further substantial changes will not be possible. This independent assessment will usually be external, independent review for both a substantial grant award from a non-commercial or government funder and ethics approval.
